# Development and Investigation of High Performance PVA/NiO and PVA/CuO Nanocomposites with Improved Physical, Dielectric and Mechanical Properties

**DOI:** 10.3390/ma15155154

**Published:** 2022-07-25

**Authors:** Faiza, Zahida Malik, Abraiz Khattak, Ahmad Aziz Alahmadi, Safi Ullah Butt

**Affiliations:** 1 School of Natural Sciences, National University of Sciences and Technology (NUST), Sector H-12, Islamabad 44100, Pakistan; faizaijaz710@gmail.com; 2 Department of Chemistry, COMSATS University Islamabad, Lahore Campus, Defence Road off Riwind Road, Lahore 51500, Pakistan; 3 High Voltage Laboratory, United States-Pakistan Center for Advanced Studies in Energy, National University of Sciences and Technology (NUST), Sector H-12, Islamabad 44000, Pakistan; safibutt541@gmail.com; 4 School of Chemistry, Faculty of Engineering and Physical Sciences, Highfield Campus, Southampton SO17 1BJ, UK; 5 Department of Electrical Engineering, College of Engineering, Taif University, KSA, P.O. Box 11099, Taif 21944, Saudi Arabia; aziz@tu.edu.sa

**Keywords:** PVA, nano-fillers, nanocomposites, optical properties, dielectric properties, mechanical properties

## Abstract

A series of polyvinyl alcohol (PVA)based composites with well dispersed nano fillers were fabricated and compared in terms of dielectric, mechanical, and optical properties. Specifically, NiO and CuO nano-fillers were utilized in a range of 0.2–0.6 wt% for thin film fabrication by solution deposition method. The characterization of nanocomposites was confirmed through FTIR, FESEM, and XRPD, whereas dielectric and mechanical properties were analyzed with respect to the filler concentrations. The bandgap of PVA/nano-filler composites reduced with an increase in NiO and CuO concentration from 0.2 to 0.6 wt%. The increase in the permittivity of the material was observed for 6 wt% of nano-fillers. The toughness of PVA/nano-filler composites was improved by increasing CuO and NiO concentration and Young’s modulus of 30.9 and 27.2 MPa for 0.6 wt% of NiO and CuO-based nanocomposite, respectively, was observed. The addition of nano-fillers showed improved optical, dielectric, and mechanical properties.

## 1. Introduction

Polyvinyl alcohol (PVA) is one of the most prominently used polymers for dielectric applications such as capacitors and high voltage AC/DC cables, etc. [1]. It has not only a high bandgap but also good dielectric strength, as well as other electrical characteristics and high mechanical endurance [2]. However, there is a constant need to enhance these required important properties as polymers are organic in nature so cannot withstand different stresses applied to them during different applications, such as leakage current, pH changes, changes in temperature, mechanical stress, and UV radiation. The backbone of the polymer is affected by these stresses, and as a result degradation in the properties of the polymer is observed [3]. To cope with this problem there is the need for fillers that do not degrade the intrinsic properties of PVA but enable it to retain its properties under these stresses. In the last few decades, several polymer nanocomposites showed excellent improvements [4,5]. Therefore, there may also be the potential in PVA nanocomposites to replace PVA in high performance applications. However, the most challenging task is to select an appropriate type, size, and concentration of fillers/nanoparticles to achieve these desired characteristics.

In dielectric applications of some nano-fillers and carbonaceous materials show amazing pseudo-capacitance [6,7]. Those with connected surface particles due to pseudo-capacitance stand in contrast to the Li ion batteries that have profoundly intercalated lattices [7]. The surface functional groups, the deformities, and the grain boundaries can fill in as the brilliant redox habitats for the charge-storing responses [8,9]. However, metal oxides (MO) are performing very well in enhancing the structural properties of the polymers, for example, the semiconductor nature and stability of nickel oxide (NiO) make it suitable for various applications, including solar photovoltaic, gas detection, electrochemical capacitance, cancer prevention agent in bio-frameworks, etc. [10]. Copper oxide (CuO) is also viewed as a promising material because of its abundance, minimal effort in preparation, chemical stability, high theoretical capacity (674 mAhg^−1^), and ecological friendliness [11,12]. Among all transition metals, due to its high specific capacitance value and its low cost, CuO is known to be the best material used up to now in a supercapacitor as electrode material [13]. It is evident in the literature that NiO and CuO show high charge storage capacity when employed as an electrode for a pseudo-supercapacitor along with KOH electrolyte. However, incorporation of filler enhances the dielectric behavior of the material but somehow compromises the mechanical properties, meaning that the optimization of the concentration and appropriate dispersion are of utmost importance.

Traditionally, composite materials are fabricated with almost 40 wt% of filler in the polymer matrix. There are recent reports on significant changes in physical properties of the materials with loadings of nano-fillers less than 10 wt% in the polymer composites [14,15]. The enhanced material properties are due to the intrinsic properties of the nano-fillers. This study aims to utilize the advantages of the tremendous surface area exhibited by nano-fillers under consideration. In the present study, we prepared films containing PVA with appropriate range of nano-filler, i.e., 0.2–0.6 wt% and examined their properties as a function of the filler content and type.

The purpose of the study was to follow an appropriate method for the synthesis of 0.2–0.6 wt% with maximum dispersion of nano-filler in the polymer matrix. The aim of the study was to investigate the changes employed in optical, dielectric, and mechanical properties by adding various concentrations of nano-fillers. Further goals were to investigate the tuning of electrical and mechanical components using nano-fillers, and their dielectric behavior, so that they may be used in different optical and electrical applications.

## 2. Sample Preparation

**Figure 1:**
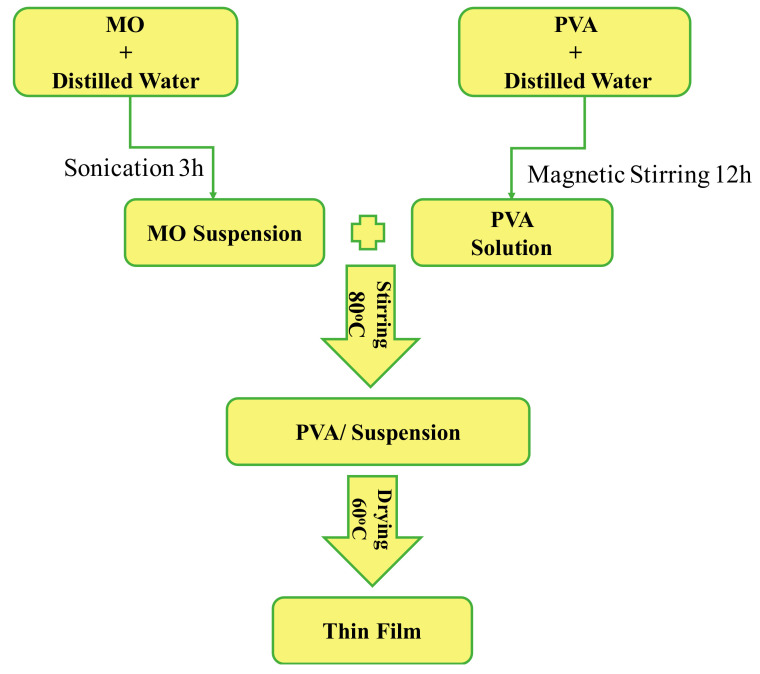
Schematic diagram for preparation of nanocomposite thin films.

The composite thin film synthesis with different concentrations of nano-filler (yield range 75–80%) in the PVA matrix was carried out by the solution deposition method, flow sheet for this procedure is given in Figure 1. The chemicals used in the synthesis were; H_2_SO_4_ (97% pure), HNO_3_ (67% pure), NiNO_3_.3H_2_O, and Cu (NO_3_)_2_.3H_2_O of analytical grade purchased from RDH (Riedel-de Haën) while PVA was of commercial grade with molecular wt. 86.09 g/mol.

NiO was prepared using sonication method. 1 molar solution of NiCl_2_.6H_2_O was prepared and subsequent dropwise addition of 2 M NaOH was carried out under constant stirring. Then the whole solution was subjected to ultra-sonication using probe sonicator for 60 min. After that the solution was filtered, washed, and dried at 250 °C to remove the residue, the product was finally calcined at 500 °C for 3 h [16] and CuO nanoparticles were synthesized using co-precipitation method where 0.02 M solution of Cu(NO_3_)_2_, 3H_2_O, and 1 mL glacial acid was refluxed and heated at 100 °C. The rapid addition of NaOH was then carried out until the pH of 6–7 was attained. A large number of black precipitates were obtained. Finally, the precipitates were washed and air dried at ambient conditions [17]. The different concentrations 0.2, 0.4, and 0.6% single phase NiO and CuO nanomaterials were added in 10% PVA solution and sonicated for 3 h after mixing in distilled water as indicated in Table 1.

These mixtures were added to the PVA solutions under constant stirring for 7 h to ensure homogeneity. Thick slurries were transferred to the glass substrates for water evaporation under vacuum drying for 17 h at 55 °C.

## 3. Instruments and Methods

### 3.1. Fourier Transform Infrared Spectroscopy (FTIR)

Fourier transform infrared spectroscopy (FTIR) was performed by using the Bruker platinum ATR model Alpha, Germany, with a spectral range of 4000–500 cm^−1^, the samples were directly placed on a nano-filler scanner.

### 3.2. X-ray Powder Diffraction (XRPD)

XRPD analysis was done by employing a STOE X-Ray diffractometer, operating at 40 kV with CuKα1 radiation within 10° < 2θ > 80° range for powder and film samples. X’pert Highscore was used to analyze the data [18].

### 3.3. Field Emission Scanning Electron Microscopy (FESEM)

Field emission scanning electron microscopy (FESEM) analysis was done on MIRA3 TESCAN Zeiss supra 55 VP for the qualitative and morphological testing of powder and film sample after Au coating.

### 3.4. UV–Vis Diffuses Reflectance Spectroscopy (UV–DRS)

Optical properties and bandgap calculations of the thin films were done through UV–Vis Diffuse Reflectance Spectroscopy (DRS) Perkin Elmer Spectrometer Lambda 950, Shelton, CT, USA, with a spectral range of 190–3300 cm^−1^.

### 3.5. Dielectric Properties

For dielectric properties measurement at room temperature, thin films were cut into the 2 mm diameter and pressed between electrodes of LCR meter of Wayne Kerr model 6500B with frequency range 100 Hz to 5 MHz.

### 3.6. Tensile Strength

Tensile testing was done using standard ASTM D638-12 of UTM (AG-X Plus) Shimadzu Japan for the samples with gauge length ~20 mm and width ~1 mm under 10 N applied force of 5 mm/min.

## 4. Results and Discussions

### 4.1. Characterization

#### 4.1.1. Fourier Transform Infrared Spectroscopy (FTIR)

FTIR spectroscopy was performed within a range of 500 to 4000 cm^−1^ in order to ensure the existence of composites.

**Figure 2:**
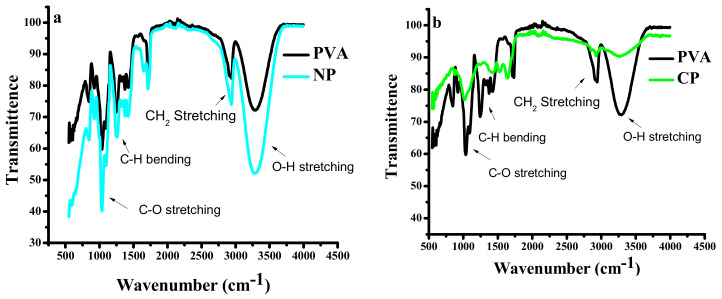

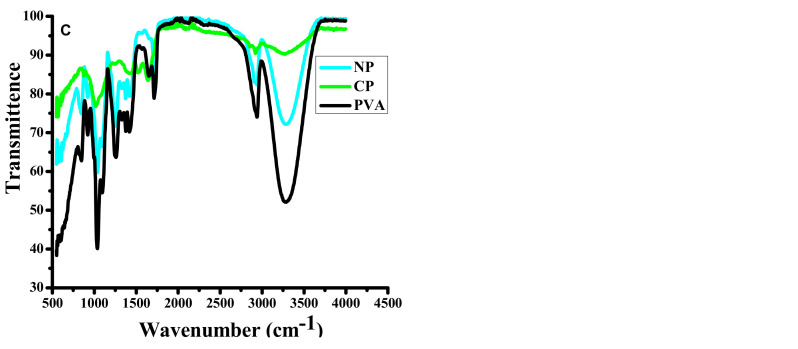
FTIR spectra, (**a**) pure PVA and PVA/NiO (NP3), (**b**) pure PVA and PVA/CuO (CP3), (**c**) comparison of PVA, NP3, and CP3.

Figure 2 shows IR spectra of PVA and PVA/nano-filler. The nano-ceramics; CuO and NiO do not exhibit transmittance between 400–4500 cm^−1^, that is why no band was observed for these materials in the spectral range under investigation. The characteristic peaks associated with PVA were observed at 1256 and 1090 cm^−1^ (C–H bending and C–O–C stretching), 1730 cm^−1^ (due to water absorption), 2940 and 2910 cm^−1^ (asymmetric stretching CH_2_) and 3330 cm^−1^ (O–H stretching) [19,20,21]. However, a decrease in transmittance due to addition of metals oxide is evident, suggesting that the filler affects the optical behavior of the polymer. It can be clearly seen from Figure 2c that the transmittance of NiO-based composite is higher than the CuO-based nanocomposite. Also, the shifting of C–O–C at 1090 and C=O band at 1700 can be seen in both NiO and CuO nanocomposites, which indicates the interaction of nanoparticles with the PVA matrix [22].

#### 4.1.2. X-ray Powder Diffraction (XRPD)

**Figure 3:**
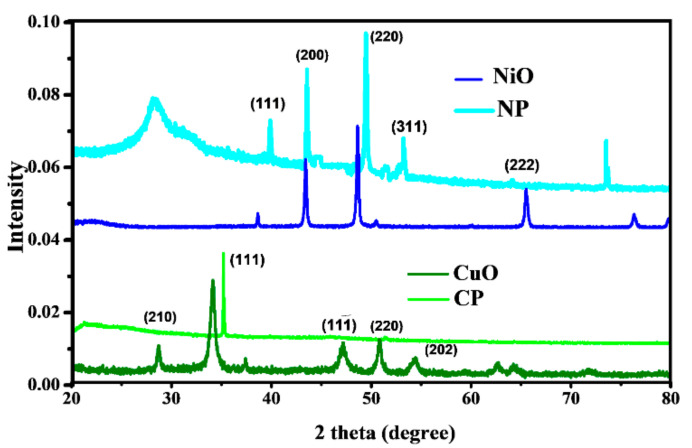
X-Ray powder diffractograms, pure NiO nano-powder and PVA/NiO(NP3) thin film, pure CuO nano-powder and PVA/CuO (CP3) thin film within 10° < 2θ > 80° at room temperature.

XRPD data of pure nano-ceramics was recorded, and crystal structures were confirmed by comparing with the literature. As shown in Figure 3, all peaks were indexed with JCPDS card no. 45-0937 and 73-1523 for NiO and CuO, respectively. The average crystallite sizes were calculated by using Scherer formula [18,23] as 34.21 and 4.98 nm for NiO and CuO, respectively, employing a shape factor of 0.9. It was seen that all the peaks of PVA, NiO, and CuO were present in spectra with a peak shift for NiO and CuO due to tilted film during analysis and also due to the lattice strain, produced in the lattice after composite formation. In the case of NiO, the diffraction peaks are observed at 20°, 37.2°, 43.2°, and 63.0°, and can be related to (111), (200), and (220) planes, respectively. It could be seen that peak at (222) shifted towards the right, which is due to a change in the mechanical parameters and crystallite size of the material as the addition of filler in the polymer increases the tensile strain, which affects the peak position [24]. Similarly, in the case of CuO, no additional impurity peak can be observed and it can be seen that only peak (111) appeared in composite, which indicates that all the CuO particles are aligned in same direction in polymer matrix. The XRD pattern can be well indexed to the monoclinic structure and the JCPDS 80-1268. The peak broadening can be attributed to the small size of the particles [25].

#### 4.1.3. Field Emission Scanning Electron Microscopy (FESEM)

**Figure 4:**
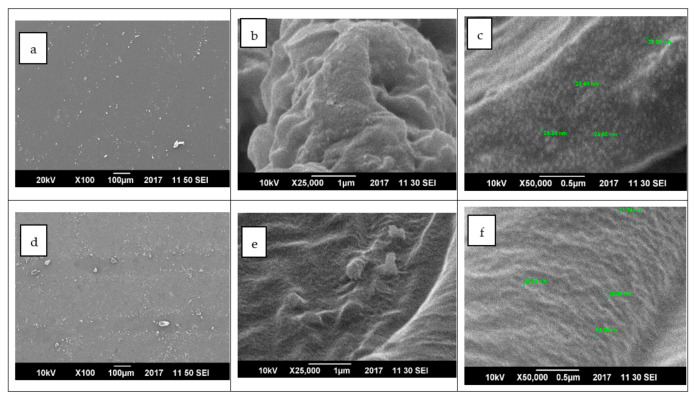
Scanning electron microscopy images of (**a**–**c**) NP3 at 100 µm, 1 µm & 0.5 µm (**d**–**f**) CP3 at at 100 µm, 1 µm & 0.5 µm.

Scanning electron microscopy was employed to check the proper dispersion of metal oxides into the polymer matrix. High resolution images of the nanocomposite with highest concentration (0.3%) for both NiO and CuO are given in Figure 4a–f. A fine dispersion of nanoparticles could be seen, however, few agglomerates of nanoparticles at 100 µm were found, which are the reason for the decrease in Young’s modulus of nanocomposite and the increase in the concentration of metal oxides [26].

### 4.2. Physical Properties

The optical, dielectric and mechanical properties of the blank PVA and the composites were investigated. UV–DRS was employed to establish the bandgap with reflectance measurements and LCR meter for dielectric properties calculations; dielectric constant, dielectric loss, tan loss, and AC conductivity.

#### 4.2.1. UV–Vis Diffusion Reflectance Spectroscopy (UV–DRS)

**Figure 5:**
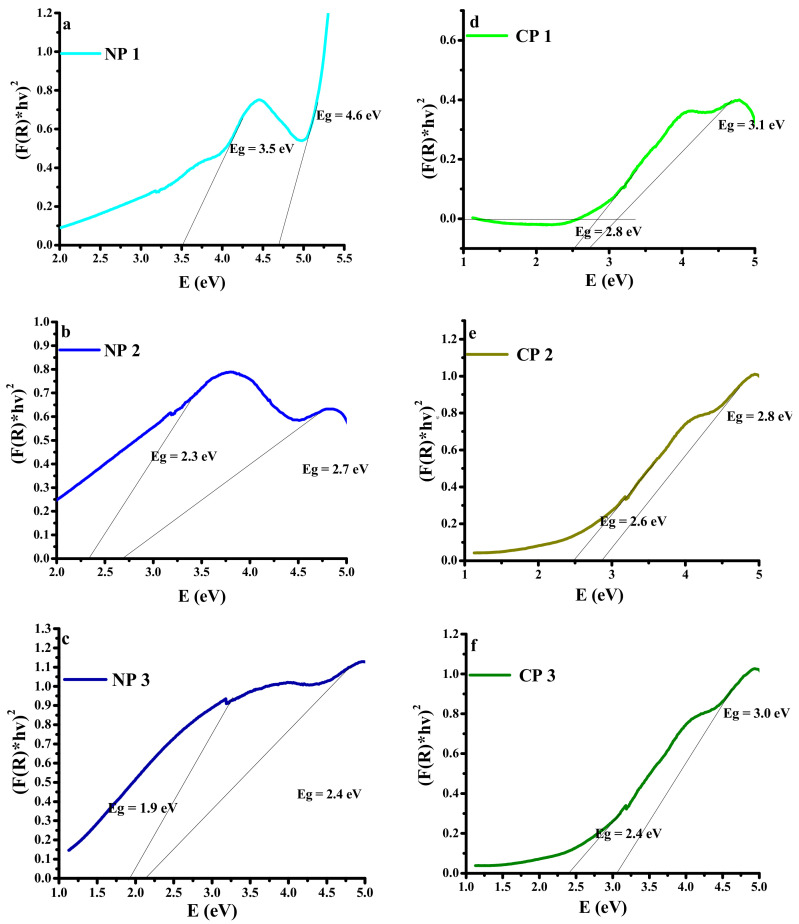
Plot of (F(R)hυ)^2^ vs. photon energy for the samples; (**a**) NP1; PVA/NiO (0.1%), (**b**) NP2; PVA/NiO (0.2%), (**c**) NP3; PVA/NiO (0.3%), (**d**) CP1; PVA/CuO (0.1%), (**e**) CP2; PVA/CuO (0.2%), (**f**) CP3; PVA/CuO (0.3%).

Optical bandgap calculations were done by UV–Vis in diffuse reflectance mode within the range, 200–1000 nm. Figure 5 shows a graph plotted between reflectance and wavelength where all the compounds show reflectance near the ultraviolet region around 300 nm. Bandgap (E_g_) was calculated by using the Kubelka–Munk Equation [27,28], given below,

(1) $$F\left(R\right)=\frac{{\left(1-R\right)}^{2}}{2R}$$

where F(R) is the reflectance coefficient and R is the reflectance. The graphs in Figure 5 were plotted between (F(R)*hυ)^2^ and energy (eV) to calculate the bandgap values [25]. It was observed that with the increase in NiO and CuO, the concentration bandgap decreased.

The optical bandgap of PVA with 0 wt% NiO and CuO is 5–6 eV [27]. It was seen that value of the bandgap for PVA decreased from 4.6 to 2.7 and 2.4 eV as 0.2, 0.4 and 0.6 wt% NiO was added to PVA, respectively. Same trend was observed in case of CuO, bandgap values for PVA after adding 0.2, 0.4 and 0.6 wt% CuO were decreased to 3.1, 2.8, and 2.4 eV, respectively. The values of energy bandgaps in different composites are dependent upon the concentrations of NiO and CuO nanomaterials. A decrease in the optical energy bandgap of nanocomposites with an increase in NiO and CuO weight as compared to that of pure PVA was observed. This trend was observed due to the ability of nano-filler particles to arrange themselves in the polymer matrix, resulting in the formation of conductive pathways in which electron hopping can occur. Such a situation leads to a shift of the valence and the conduction bands as well as an enhancement of carrier–carrier interaction. Due to the high concentration of carrier in valence and conduction bands, the bandgap is reduced. The bandgap of the PVA-based nanocomposite can be tuned according to need by varying the concentration of nanomaterials, as listed in Table 2. Table 2 and Figure 4 show the bandgaps for the nano-filler and the PVA in each composite.

The change seen in the bandgap shows that by addition of the nano-filler the energy states of the pure PVA are modified, creating the localized states in forbidden band due to which the Fermi level might change, and these states act as recombination and trapping centers. Therefore, due to low energy transitions, the opteoelectrical conductivity of the nanocomposites was enhanced [28].

#### 4.2.2. Dielectric Properties

To anticipate the application of prepared nanocomposites, the dielectric properties were measured at room temperature using the LCR meter. The different parameters investigated are dielectric constant, dielectric loss, tangent loss, and AC conductivity. The dielectric parameter could be represented as follows,

(2) $$\;\varepsilon^{*}=\varepsilon^{\prime }-\;i\varepsilon^{\prime \prime }$$

where $\varepsilon^{\prime }$ is the real part and $i\varepsilon^{\prime \prime }\;\mathrm{is}\;\mathrm{the}\;\mathrm{imaginary}\;\mathrm{part}$ out of which the real part can be calculated from the formula given by,

(3) $$\varepsilon^{\prime }=\frac{C\;d}{{A\;\varepsilon }_{o}}$$

**Figure 6:**
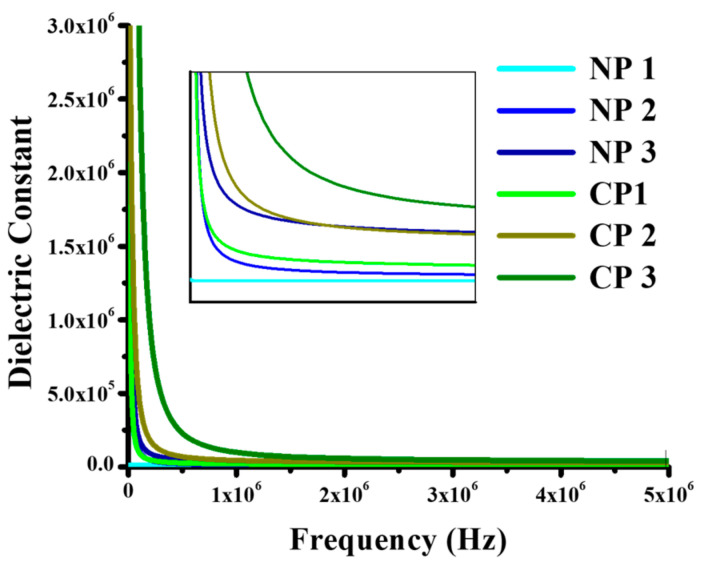
The graphs between dielectric constant verses ln F(Hz) for the samples; NP1; PVA/NiO (0.01 g), NP2; PVA/NiO (0.02 g), NP3; PVA/NiO (0.03 g), CP1; PVA/CuO (0.01 g), CP2; PVA/CuO (0.02 g), CP3; PVA/CuO (0.03 g).

This part explains the ability to transfer the charges or the ability of a material to store the charges. Where ‘C’ is capacitance, d refers to the thickness in mm, ‘A’ refers to the surface area in m^2^, and $\varepsilon_{o}$ is the permittivity of the space $\;\left({\;\varepsilon }_{o}=8.854\times 10^{-12}{\mathrm{Fm}}^{-1}\right)$ , all these parameters are responsible for the charge-storing ability of the materials [30]. Dielectric constant is dependent upon the ability of a material to separate charges and the extent to which it can oppose the movement of charges. As the amount of NiO and CuO increases, bandgap decreases, and charges become mobile. The charge-storing ability of the material then decreases, resulting in a decrease in dielectric constant. Dielectric constant is also dependent on grain boundaries that are created within the material when the external electrical charge applied. The high dielectric constant values were observed in case of NiO and CuO-doped thin films due to high polarization at low frequency, and as the frequency increases, the previously formed dipoles fail to arrange themselves with fluctuating current and heat losses take place. Subsequently, a very small amount of charge is stored so dielectric constant decreases and, as can be seen in Figure 6 at highest measured frequency, it becomes constant. From Figure 6, it can be seen that dielectric constant increased with the higher amount of nano-filler.

**Figure 7:**
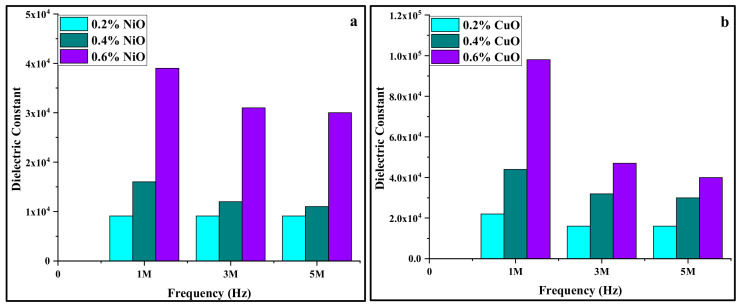
Bar graph showing trend of dielectric constant (**a**) wt% of NiO (**b**) wt% of CuO in PVA with variation in frequency.

At low frequencies dipoles follow the electric field, but at higher frequencies dielectric properties vary and dielectric constant decreases as dipole begin to lag behind the field [31]. As the amount of NiO increased from 0.2 to 0.4 and 0.6 wt%, their dielectric constant values changed from 9.1 × 10^3^, 1.6 × 10^4^, and 3.9 × 10^4^ F/m at 1 × 10^6^ Hz, to 9.1 × 10^4^, 1.2 × 10^4^, and 3.1 × 10^4^ F/m at 3 × 10^6^ Hz, and 9.1 × 10^3^, 1.1 × 10^4^, and 3.0 × 10^4^ F/m at 5 × 10^5^ Hz. The same trend was observed for CP1, CP2, and CP3, as their dielectric constant values were recorded as 2.2 × 10^4^, 4.4 × 10^4^, and 9.8 × 10^4^ F/m at 1 × 10^6^ Hz, 1.6 × 10^4^, 3.2 × 10^4^, and 4.7 × 10^4^ F/m at 3 × 10^6^ Hz, and 1.6 × 10^4^, 3.0 × 10^4^, and 4.0 × 10^4^ F/m at 5 × 10^5^ Hz. This trend is shown below in the bar graphs (Figure 7). It can be seen that as the amount nano-filler increased, the charge-storing ability of the material also increased, and CuO-reinforced composites have shown better results as compared to NiO-reinforced composites.

**Figure 8:**
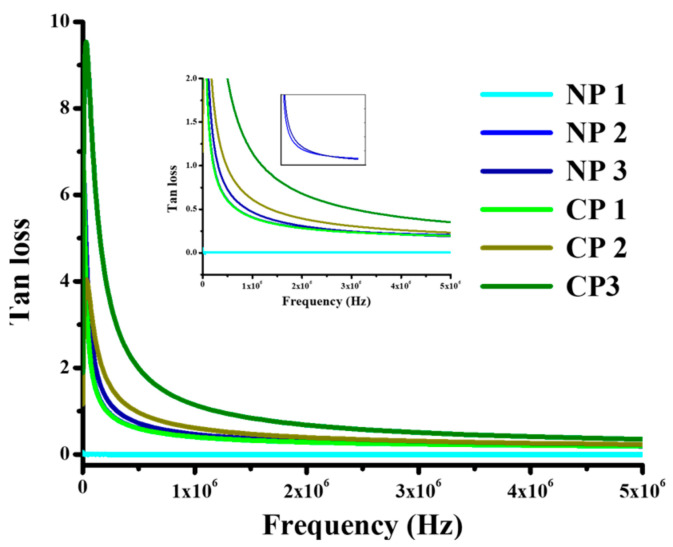
Tan loss, NP1; PVA/NiO (0.01 g), NP2; PVA/NiO (0.02 g), NP3; PVA/NiO (0.03 g), CP1; PVA/CuO (0.01 g), CP2; PVA/CuO (0.02 g), CP3; PVA/CuO (0.03 g).

The heat loss is expressed in terms of tan loss and it can be expressed mathematically by the following formula,

(4) $$\tan\delta \;=\frac{\varepsilon^{\prime \prime }}{\varepsilon^{\prime }\;}$$

where $\varepsilon^{\prime \prime }$ is the imaginary part of permittivity (dielectric constant) and $\varepsilon^{\prime }$ is the real part of permittivity. Tan loss (tan δ) is a relative measure of energy loss [32]. Values observed in case of tan loss were low and its value periodically increased as the amount of nano-filler increased in PVA as shown in Figure 6. As discussed in Section 4.2.1, the decrease in bandgap with nano-filler addition of 0.5 eV results in higher conductivity and the movement of charges leads to greater tan *δ* due to interfacial polarization of charges accumulated at interfaces. It is evident from Figure 7 that heat loss increased as the amount of filler increased, which can be attributed to increased polarization and ohmic losses. From Figure 8 it can be seen that tan *δ* value decreases with the increase in frequency. As the high frequency atom did not show rapid electron transfer, the dielectric loss value also decreases. This is due to the fact that, at a higher frequency, less energy is consumed by the hopping of electrons and heat loss also is also reduced at a higher frequency [33].

Tan loss values are higher in low frequency ranges. Increased filler content gives rise to a conductive pathway by decreasing bandgap as confirmed previously by bandgap calculations. In this way, electrons have a greater mean free path at which they move in every cycle of the alternating field.

AC conductivity of the material is its ability to allow the passage of current. When the external field is applied to the materials, they align themselves and their hopping frequency resonates with the external frequency and it can be measured as in Equation (5) [30].

(5) $$\sigma_{\mathrm{ac}}=2\pi f\varepsilon^{\prime }\varepsilon_{o}\tan\delta \;$$

**Figure 9:**
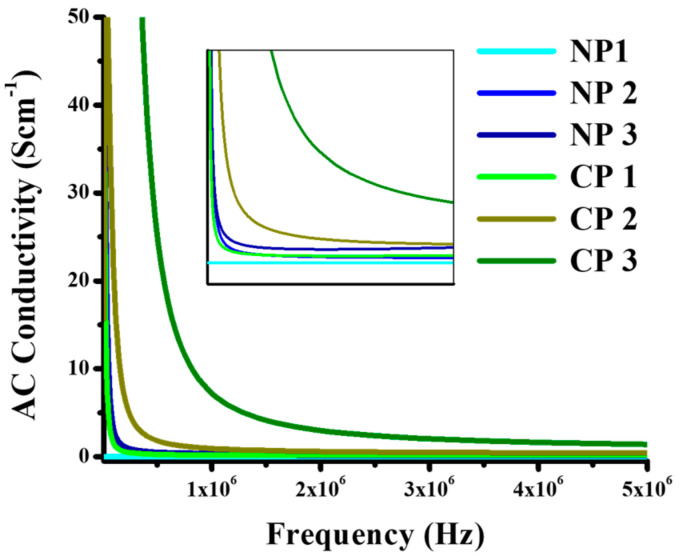
AC conductivity, PVA/NiO (0.2 wt%), PVA/NiO (0.4 wt%), PVA/NiO (0.6 wt%), PVA/CuO (0.2 wt%), PVA/CuO (0.4 wt%), PVA/CuO (0.6 wt%).

Figure 9 for AC conductivity of NiO and CuO-reinforced PVA composite showed that AC conductivity increases as the amount of nano-filler in PVA increases which is due to the fact that as nano-filler is added, conductive pathways are formed that cause a decrease in bandgap and an increase in conductivity. Charge carriers easily align themselves according to the externally applied field, and it was observed that conductivity decreases as frequency increases for the reason that at higher frequency, charge carriers do not get enough time to arrange themselves and they move randomly, meaning that AC conductivity decreases [31]. As the bandgap of composites decreases with the increase in the amount of nano-filler, so the material with low bandgap showed the high conductivity, and with an increase in bandgap, conductivity decreases. AC conductivity values for 0.2, 0.4, and 0.6 wt% of NiO were 0.04, 0.20, and 0.30 S cm^−1^ at 1 × 10^6^ Hz, 0.04, 0.10, and 0.28 S cm^−1^ at 3 × 10^6^ Hz, and 0.04, 0.09, and 0.30 S cm^−1^ at 5 × 10^6^ Hz, respectively. A similar trend was observed in the case of CuO, AC conductivity values for 0.2, 0.4, and 0.6 wt% of NiO were 0.20, 0.90, and 6.90 S cm^−1^ at 1 × 10^6^ Hz, 0.10, 0.40, and 1.90 S cm^−1^ at 3 × 10^6^ Hz, and 0.17, 0.40, and 1.40 S cm^−1^ at 5 × 10^6^ Hz, respectively. It is evident from the results that composites perform best at lower frequencies up to 1 MHz.

#### 4.2.3. Tensile Strength

Nano-fillers play important roles in tailoring the mechanical properties of polymers despite being used in wt < 10 wt% [34,35,36,37]. Rigid inorganic nanoparticles with a smaller aspect ratio are quite promising for reinforcing and toughening materials for the PVA matrix. The mechanical properties of NiO and CuO nano-fillers in PVA matrix composite films were investigated by using the universal testing machine and by drawing a stress vs. strain graph and different parameters; Young’s modulus (E), elastic limit (S_y_), ultimate tensile strength (UTS), total strain (e_total_), and elastic strain (e_elastic_) were calculated [38]. The high brittleness of nano-fillers expected to control the high elasticity of the polymer and enhance its mechanical properties. By using a stress vs. strain graph, the mechanical properties parameters were calculated. It was seen that high elastic modulus of PVA is balanced by brittle metal oxide materials as shown in Table 3.

The elastic limits tended to decrease with the nano-filler loadings, from 2.1 to 1.2 for NiO 0.2 to 0.6 wt%, respectively. The same can be concluded for the elastic limit in the case of CuO nano-filler. The values of total strain and elastic strain given in Table 3 indicate the need to consider averaged values due to the close and nonuniform trends. In the nano-filler loadings of the range under consideration there is a 63% and 75% decrease in the values of Young’s modulus for NiO and CuO thin films, respectively. Elastic limits decreased from 34.4 (Pure PVA) to 1.63 and 3.96 for NiO and CuO, respectively. In both cases, elastic limits are far different as compared to that of the pure PVA but NiO ~ 2 has shown half that of the CuO, i.e., ~4. The values for Ultimnate Tensile Strength UTS decreased from 95 MPa (for pure PVA) to 4.18 and 0.55 MPa for NiO and CuO, respectively. There are similar reports on thermoplastics and elastomers loaded with nano-silica and calcium carbonate, simultaneously improving stiffness and toughness [39,40,41]. The rigid nanoparticles also improve the tensile ductility of the PMMA significantly [42,43,44].

The composites with 0.2 wt% NiO have superior mechanical properties (E = 22.7 MPa and UTS = 4.9 MPa) with greater total and elastic strain values, i.e., 0.34 and 0.26, respectively, as compared to those of the composites with CuO nano-filler and other NiO concentrations. Due to presence of nano-filler, high dielectric constant was achieved and the elastic nature of PVA was overcome, keeping in view their application in the elastic electronics [29,44,45,46].

## 5. Conclusions

Different compositions of NiO and CuO-based PVA nanocomposites were prepared and the effect of fillers and their concentration was analyzed for physical, mechanical, and dielectric properties. It was observed that the bandgap of nanocomposites decreased with an increase in filler concentration for both NiO and CuO fillers with CP1, CP2, and CP3, expressing 3.1, 2, and 3.0 eV, respectively. Similarly, NP1, NP2 and NP3 exhibited a bandgap of 4.6, 2.7, and 2.4, respectively. It is evident that a higher reduction in bandgap was seen for CuO-based nanocomposites. The tensile strength of nanocomposites decreased with an increase in the filler concentration owing to the fact that inorganic oxide fillers reduce the elasticity while increasing the hardness of polymer materials. The investigation of dielectric properties demonstrated that composites with the highest concentrations of fillers exhibited the highest dielectric constant, hence expressing greater charge-storing ability. NP3 expressed dielectric constant, loss, and AC conductivity values of 9.1 × 10^3^, 1.1, and 6.9 S cm^-1^, respectively, at 1.1 × 10^6^ Hz, while CP3 exhibited 2.2 × 10^4^, 0.42, and 0.3 S cm^−1^, respectively. The overall result showed that by incorporation of MOs in the PVA matrix we can tune the bandgap and improve the dielectric behavior of the materials, retaining its high Young’s modulus so that they might be used in different optical and electrical applications.

## Figures and Tables

**Table 1:** List of samples with different amounts of nano-fillers in 10% PVA solution.

Sr. #	Sample Code	NiO (wt%)	CuO (wt%)
1	CP 1	-	0.2
2	CP 2	-	0.4
3	CP 3	-	0.6
4	NP 1	0.2	-
5	NP 2	0.4	-
6	NP 3	0.6	-

**Table 2:** Calculation of bandgap of all the prepared composites using Kubelka–Munk Equation [29].

Sr. No.	Sample	Bandgap (eV)	
		Nano-Filler	PVA
1	NP 1	3.5	4.6
2	NP 2	2.3	2.7
3	NP 3	1.9	2.4
4	CP 1	2.8	3.1
5	CP 2	2.6	2.8
6	CP 3	2.4	3.0

**Table 3:** Mechanical properties of PVA and nanocomposite thin films.

Code	Sample Compositions	Young’s Modulus (E)		Elastic Limit (S_y_) MPa	* Ultimate Tensile Strength (UTS) MPa	Total Strain (e_total_)	Elastic Strain (e_elastic_)
		MPa	Difference				
0	PVA@0 wt% NiO	71.5	-	34.4	95.4	0.37	0.32
NP 1	PVA@0.2 wt% NiO	22.7	68%	2.1	4.9	0.34	0.26
NP 2	PVA@0.4 wt% NiO	25.7	64%	1.6	2.5	0.26	0.13
NP 3	PVA@0.6 wt% NiO	30.9	56.7%	1.2	5.14	0.5	0.4
**Averaged values for** **PVA@ NiO nano-filler**		**26.43**	**63.03%**	**1.63**	**4.18**	**0.366**	**0.26**
CP 1	PVA@0.2 wt% CuO	8.2	88.53%	1.09	5.13	0.60	0.44
CP 2	VA@0.4 wt% CuO	18.9	73.56%	0.37	1.7	0.62	0.53
CP 3	PVA@0.6 wt% CuO	27.2	61.95%	1.53	5.06	0.47	0.39
**Averaged values for** **PVA@ CuO nano-filler**		**18.1**	**74.68%**	**3.96**	**0.55**	**0.44**	**1.16**

* Maximum tensile strength a material can withstand before breaking while pulled/stretched.

## Data Availability

The data presented in this study are available on the request from the corresponding author.

## References

[1] Liu S.-H., Lai Y.-C., Lin C.-W. (2018). Enhancement of power generation by microbial fuel cells in treating toluene-contaminated groundwater: Developments of composite anodes with various compositions. Appl. Energy.

[2] Reddy P.L., Deshmukh K., Chidambaram K., Ali M.M.N., Sadasivuni K.K., Kumar Y.R., Lakshmipathy R., Pasha S.K.K. (2019). Dielectric properties of polyvinyl alcohol (PVA) nanocomposites filled with green synthesized zinc sulphide (ZnS) nanoparticles. J. Mater. Sci. Mater. Electron..

[3] Khattak A., Amin M., Iqbal M., Abbas N. (2018). Life estimation and analysis of dielectric strength, hydrocarbon backbone and oxidation of high voltage multi stressed EPDM composites. Mater. Res. Express.

[4] El-Molla S., Mansour A.F., Hammad A. (2017). Enhancement of Fluorescence and Photostability Based on Interaction of Fluorescent Dyes with Silver Nanoparticles for Luminescent Solar Concentrators. J. Nanomater..

[5] Ramanathan T., Abdala A.A., Stankovich S., Dikin D.A., Herrera-Alonso M., Piner R.D., Adamson D.H., Schniepp H.C., Chen X., Ruoff R.S. (2008). Functionalized graphene sheets for polymer nanocomposites. Nat. Nanotechnol..

[6] Yu B. (2001). NANO-FILLERS Transistor with Assisted-Gates and Ultra-Shallow “Psuedo” Source and Drain Extensions for Ultra-Large-Scale Integration. U.S. Patent.

[7] Zhang L.L., Zhao X.S. (2009). Carbon-based materials as supercapacitor electrodes. Chem. Soc. Rev..

[8] Zhou L., Li C., Liu X., Zhu Y., Wu Y., van Ree T. (2018). Nano-Fillers in Supercapacitors.

[9] Liu C., Yu Z., Neff D., Zhamu A., Jang B.Z. (2010). Graphene-Based Supercapacitor with an Ultrahigh Energy Density. Nano Lett..

[10] Saikia J.P., Paul S., Konwar B.K., Samdarshi S.K. (2010). Nickel oxide nanoparticles: A novel antioxidant. Colloids Surfaces B Biointerfaces.

[11] Zhi M., Xiang C., Li J., Li M., Wu N. (2013). Nanostructured Carbon–nano-filler Composite Electrodes for Supercapacitors: A Review. Nanoscale.

[12] Zhang X., Shi W., Zhu J., Kristal D.J., Zhao W., Lalia B.S. (2011). High-power and High-Energy-Density Flexible Pseudocapacitor Electrodes Made from Porous CuO Nanobelts and Single-walled Carbon Nanotubes. ACS Nano.

[13] Liu Y., Cao X., Jiang D., Jia D., Liu J. (2018). Hierarchical CuO nanorod arrays in situ generated on three-dimensional copper foam via cyclic voltammetry oxidation for high-performance supercapacitors. J. Mater. Chem. A.

[14] Pradhan B., Setyowati K., Liu H., Waldeck D.H., Chen J. (2008). Carbon Nanotube−Polymer Nanocomposite Infrared Sensor. Nano Lett..

[15] Yang X., Li L. (2010). Synthesis and characterization of layer-aligned poly(vinyl alcohol)/graphene nanocomposites. Polymer.

[16] Duraisamy N., Numan A., Fatin S.O., Ramesh K., Ramesh S. (2016). Facile sonochemical synthesis of nanostructured NiO with different particle sizes and its electrochemical properties for supercapacitor application. J. Colloid Interface Sci..

[17] Zhu J., Li D., Chen H., Yang X., Lu L., Wang X. (2004). Highly dispersed CuO nanoparticles prepared by a novel quick-precipitation method. Mater. Lett..

[18] Patel V.B., Theron G., Lenders L., Matinyena B., Connolly C., Singh R., Coovadia Y., Ndung’U T., Dheda K. (2013). Diagnostic Accuracy of Quantitative PCR (Xpert MTB/RIF) for Tuberculous Meningitis in a High Burden Setting: A Prospective Study. PLoS Med..

[19] Davar F., Fereshteh Z., Salavati-Niasari M. (2009). Nanoparticles Ni and NiO: Synthesis, Characterization and Magnetic Properties. J. Alloys Compd..

[20] Mansur H., Sadahira C.M., Souza A.N., Mansur A. (2008). FTIR spectroscopy characterization of poly (vinyl alcohol) hydrogel with different hydrolysis degree and chemically crosslinked with glutaraldehyde. Mater. Sci. Eng. C.

[21] Prabhudass J.M., Palanikumar K., Natarajan E., Markandan K. (2022). Enhanced Thermal Stability, Mechanical Properties and Structural Integrity of MWCNT Filled Bamboo/Kenaf Hybrid Polymer Nanocomposites. Materials.

[22] Patterson A.L. (1939). The Scherrer Formula for X-ray Particle Size Determination. Phys. Rev..

[23] Monshi A., Foroughi M.R., Monshi M.R. (2012). Modified Scherrer Equation to Estimate Nano-fillerre Accurately Nano-crystallite Size Using XRD. World J. Nano Sci. Eng..

[24] Kouklin N., Tzolov M., Straus D., Yin A., Xu J.M. (2004). Infrared absorption properties of carbon nanotubes synthesized by chemical vapor deposition. Appl. Phys. Lett..

[25] Rao J.K., Raizada A., Ganguly D., Mankad M.M., Satyanarayana S.V., Madhu G.M. (2015). Enhanced mechanical properties of polyvinyl alcohol composite films containing copper oxide nanoparticles as filler. Polym. Bull..

[26] Yang L., Kruse B. (2004). Revised Kubelka–Munk Theory. I. Theory and Application. JOSA A.

[27] Abdullah G., Aziz S.B., Omer K.M., Salih Y.M. (2015). Reducing the Optical Bandgap of Polyvinyl alcohol (PVA) Based Nanocomposite. J. Mater. Sci. Mater. Electron..

[28] Aslam M., Kalyar M.A., Raza Z.A. (2021). Fabrication of nano-CuO-loaded PVA composite films with enhanced optomechanical properties. Polym. Bull..

[29] Mitlin D., Ding J. (2020). Hydrogel Derived Carbon for Energy Storage Devices. U.S. Patent.

[30] Tiuri M., Sihvola A., Nyfors E., Hallikaiken M. (1984). The complex dielectric constant of snow at microwave frequencies. IEEE J. Ocean. Eng..

[31] Da Silva A.B., Arjmand M., Sundararaj U., Bretas R.E.S. (2014). Novel composites of copper nanowire/PVDF with superior dielectric properties. Polymer.

[32] Rao J.K., Raizada A., Ganguly D., Mankad M.M., Satayanarayana S.V., Madhu G.M. (2015). Investigation of structural and electrical properties of novel CuO–PVA nanocomposite films. J. Mater. Sci..

[33] Long Y., Shanks R.A. (1996). PP–elastomer–filler hybrids. I. Processing, microstructure, and mechanical properties. J. Appl. Polym. Sci..

[34] Bartczak Z., Argon S.A., Cohen R.E., Weinber M. (1999). Toughness mechanism in semi-crystalline polymer blends: II. High-density polyethylene toughened with calcium carbonate filler particles. Polymer.

[35] Misra R.D.K., Nerikar P., Bertrand K., Murphy D. (2004). Some aspects of surface deformation and fracture of 5–20% calcium carbonate-reinforced polyethylene composites. Mater. Sci. Eng..

[36] Popovics S. (1973). A numerical approach to the complete stress-strain curve of concrete. Cem. Concr. Res..

[37] Luyt A.S., Dramićanin M.D., Antić Ž., Djoković V. (2009). Morphology, mechanical and thermal properties of composites of polypropylene and nanostructured wollastonite filler. Polym. Test..

[38] Musto P., Ragosta G., Scarinzi S., Mascia L. (2004). Microstructural features, diffusion and molecular relaxations in polyimide/silica hybrids. Polymer.

[39] Lin Y., Chen H., Chan M.C. (2011). Nucleating effect of calcium stearate coated CaCO_3_ nanoparticles on polypropylene. J. Colloid Interface Sci..

[40] Falcaro P., Ricco R., Yazdi A., Imaz I., Furukawa S., Maspoch D., Doonan C.J. (2016). Application of metal and metal oxide nanoparticles@ MOFs. Coord. Chem. Rev..

[41] Petrovic Z.S., Javni I., Waddon A., Banhegyi G. (2000). Properties of bulk-polymerized thermoplastic polyurethane nanocomposites. Polymer.

[42] Siegel R., Chang S., Ash B., Stone J., Ajayan P., Doremus R., Schadler L. (2001). Mechanical behavior of polymer and ceramic matrix nanocomposites. Scr. Mater..

[43] Ash B.J., Rogers D.F., Wiegand C.J., Schadler L.S., Siegel R.W., Benicewicz B.C., Apple T. (2002). Preparation and characterization of poly (lactic acid)-grafted TiO_2_ nanoparticles with improved dispersions. Appl. Surf. Sci..

[44] Ash B.J., Siegel R.W., Schadler L.S. (2004). Polymer nanocomposites: A small part of the story. Macromolecules.

[45] Zhang P., Wang F., Yu M., Zhuang X., Feng X. (2018). Two-dimensional materials for miniaturized energy storage devices: From individual devices to smart integrated systems. Chem. Soc. Rev..

[46] Kumar Y.R., Deshmukh K., Ali M.M.N., Abhijay G., Al-Onazi W.A., Al-Mohaimeed A.M., Pasha S.K. (2021). Structure, morphology and modelling studies of polyvinylalcohol nanocomposites reinforced with nickel oxide nanoparticles and graphene quantum dots. Environ. Res..

